# Cardiopulmonary exercise testing as a predictor of complications

**DOI:** 10.1308/rcsann.2014.96.1.86

**Published:** 2014-01

**Authors:** F Yanni, LH Moyes, CJ McCaffer, RC Carter

**Affiliations:** Doncaster and Bassetlaw Hospitals NHS Foundation Trust,UK

I read your article with interest. However, a sentence under the subheading ‘Cardiopulmonary exercise testing’ in the methods section (*‘Two key measurements can be determined from these data*, [oxygen uptake at peak exercise and anaerobic threshold], *indicating the point at which anaerobic metabolism is inadequate to maintain high energy phosphate production* […]’) does not coincide with the principle of cardiopulmonary exercise testing. I think you mean ‘indicating the point at which aerobic metabolism is inadequate…’ (The word ‘anaerobic’ should be corrected to ‘aerobic’.)

